# Isoferulic acid suppresses *Escherichia coli* biofilm formation via LuxS/AI-2 quorum sensing inhibition and synergizes with fosfomycin

**DOI:** 10.3389/fmicb.2026.1837128

**Published:** 2026-06-01

**Authors:** Yubin Bai, Zhijin Zhang, Jing Xu, Rongbin Hu, Zixuan Shang, Xiaojuan Wei, Weiwei Wang, Bing Li, Zhen Zhu, Jiyu Zhang

**Affiliations:** 1 Key Laboratory of New Animal Drug Project of Gansu Province, Key Laboratory of Veterinary Pharmaceutical Development of the Ministry of Agriculture and Rural Affairs, New Veterinary Drugs Engineering Research Center of Gansu Province, Key Laboratory of New Veterinary Drugs Engineering of CAAS, Laboratory Animal Science Data Center of Gansu Province, Lanzhou Institute of Husbandry and Pharmaceutical Sciences of CAAS, Lanzhou, Gansu, China; 2 College of Life Science and Food Engineering, Hebei University of Engineering, Handan, Hebei, China

**Keywords:** biofilm, *Escherichia coli*, isoferulic acid, quorum sensing system, synergistic antibacterial

## Abstract

*Escherichia coli* (*E. coli*) O157:H7, a highly virulent foodborne pathogen, poses a substantial threat to public and veterinary health. Its ability to form robust biofilms significantly amplifies virulence and confers resistance to conventional antibiotics, often leading to therapeutic failure. In this study, we employed a genetically engineered LuxS-eGFP reporter strain to screen for compounds targeting the LuxS/AI-2 quorum sensing (QS) system—a key regulator of biofilm formation. Our investigation identified Isoferulic Acid (IFA) as a potent inhibitor of this pathway. IFA effectively suppressed *de novo* biofilm formation in *E. coli* O157:H7 without exerting bactericidal effects or impairing general metabolic activity, and it also demonstrated efficacy in dispersing pre-established mature biofilms. Furthermore, we established a novel combinatorial therapeutic strategy by integrating IFA with the antibiotic sodium fosfomycin. This combination exhibited a marked synergistic effect, significantly enhancing antibacterial efficacy against *E. coli* both *in vitro* and *in vivo*. These results substantiate that IFA possesses significant antibiofilm activity and a unique capacity to potentiate antibiotic action, thereby offering a promising new avenue for combating recalcitrant *E. coli* infections.

## 1 Introduction

Shiga toxin-producing *Escherichia coli* (*E. coli*) O157:H7 is a highly pathogenic foodborne bacterium that can cause severe clinical symptoms, including hemorrhagic diarrhea and potentially fatal hemolytic uremic syndrome (Riley et al., 1983; Gyles, 2007). A defining feature of this serotype is its remarkably low infectious dose—ingestion of fewer than 10 viable cells can initiate successful colonization and rapid proliferation in the host, culminating in disease onset (Paton and Paton, 1998). Low-dose infectivity presents a formidable challenge to current food safety monitoring and public health prevention systems (Holzer et al., 2025). The virulence of *E. coli* O157:H7 is intrinsically linked to its physiological state (Gelalcha et al., 2022). In natural and host environments, the bacteria exists primarily in two forms: the planktonic state and the biofilm state, with the latter playing a pivotal role in multiple pathogenic mechanisms (Sharifi et al., 2018; Flemming et al., 2016). Substantial evidence demonstrates that the *E. coli* O157:H7 strain exhibits robust adhesion and biofilm formation capabilities on abiotic and biotic substrates (Ryu et al., 2004). Crucially, biofilm-embedded cells exhibit significantly enhanced antibiotic resistance to antimicrobial agents and host immune defenses, which not only leads to persistent and recurrent clinical infections with reduced treatment efficacy, but also markedly elevates the risk of pathogen colonization and transmission in clinical settings and on medical devices, posing a severe threat to clinical management and public health (Mah and O’Toole, 2001; Kannappan et al., 2017; Li et al., 2023). Given that over 80% of persistent bacterial infections are biofilm-associated, the recalcitrant biofilms formed by *E. coli* O157:H7 are of particular concern in clinical settings, where they contribute to refractory infections and represent a significant threat to patient outcomes and public health security (Attila et al., 2008; Kintz et al., 2019; Mok et al., 2022).

Biofilm is a structured community formed by microorganisms through the secretion of exopolysaccharide (EPS) and other matrices. This robust three-dimensional architecture facilitates the tenacious adherence of microbial consortia to a diverse range of biotic and abiotic surfaces (Flemming et al., 2016; Gupta et al., 2016; Wang et al., 2020). The development of this structured mode of existence is not a stochastic process but is meticulously governed by the bacterial quorum sensing (QS) system (Juszczuk-Kubiak, 2024). Functioning as a core cell-to-cell communication network, QS enables bacteria to synchronize gene expression in response to population density, thereby regulating critical pathogenic phenotypes such as biofilm formation, virulence factor secretion, motility, and antibiotic resistance (Miranda et al., 2022; Ghanem et al., 2023; Khider et al., 2019; Bhuiyan, 2025; Haque et al., 2024). Within the QS framework, autoinducer-2 (AI-2) is recognized as a universal signaling molecule that facilitates interspecies communication (Waters and Bassler, 2005; Choudhary and Schmidt-Dannert, 2010; Reading and Sperandio, 2006; Cui and Kim, 2024; Sun et al., 2020). AI-2 is synthesized from S-adenosylmethionine (SAM) via the LuxS enzyme, producing the precursor DPD, which spontaneously rearranges (Wang et al., 2020; Fernandes and Bentley, 2009; Hu et al., 2018; Thompson et al., 2015). In *E. coli*, the periplasmic protein LsrB captures AI-2, and the LsrACD transporter internalizes it. Inside the cell, AI-2 is phosphorylated by LsrK. Phospho-AI-2 then inactivates the repressor LsrR, initiating a positive feedback loop that upregulates the Lsr operon. The signal is terminated by degradation via LsrG and LsrF (Zuo et al., 2019; Pereira et al., 2012; Xavier and Bassler, 2005). Additionally, biofilms formation is closely related to bacterial adhesion and motility. The csgD gene, a master regulator, orchestrates the biosynthesis of curli fimbriae—amyloid fibers that are indispensable for initial surface attachment and intercellular adhesion (Serra et al., 2016). Concurrently, flagella-mediated motility enables surface exploration and colonization (Nakamura and Minamino, 2019). Flagellar assembly is a hierarchical process initiated by the master regulator flhDC, which activates the expression of class II flagellar genes (Ma et al., 2022; Takada et al., 2023). Among these, fliC encodes flagellin, the principal structural protein of the filament, while fliN codes for a component of the C-ring switch complex (Schwan et al., 2020; Kaur and Taneja, 2024). The flagellar motor, powered by a transmembrane proton motive force, is driven by the stator protein MotA (Kaur and Taneja, 2024).

Given the critical role of biofilms in bacterial antibiotic resistance and persistent infections, investigations into the molecular mechanisms governing their assembly and structural robustness have yielded transformative insights for anti-infective research (Rather et al., 2021; Condren et al., 2020; Hawas et al., 2022). Strategic interventions aimed at suppressing biofilm genesis or compromising their architectural integrity not only potentiate the efficacy of conventional antibiotics but also mitigate the propensity for resistance development (Roy et al., 2018; Rao et al., 2021). Consequently, the pursuit of high-performance novel biofilm inhibitors—especially those exhibiting synergistic activity with established antimicrobial agents—represents a frontier strategy for addressing recalcitrant bacterial infections in clinical practice.

Natural products have emerged as ideal candidates for the development of novel biofilm inhibitors due to their wide availability, high safety, and low propensity to induce drug resistance (Er-Rahmani et al., 2024; Bhowmik et al., 2025). Isoferulic Acid (IFA, The structural formula is shown in Supplementary Figure S1), a phenolic acid compound, serves as the active component in the roots of the traditional Chinese medicinal herb *Platycodon grandiflorus* (Ji et al., 2020). Previous studies have confirmed that this natural small molecule compound possesses antiviral (Chen et al., 2024), anti-hematologic malignancy (Long et al., 2020), and antioxidant properties (Meeprom et al., 2013; Kalinowska et al., 2021). However, its potential to inhibit bacterial biofilm formation remains unexplored. In this study, we constructed a LuxS-eGFP reporter strain to screen for IFA as a specific inhibitor of the LuxS/AI-2 quorum sensing (QS) pathway. We systematically evaluated its anti-biofilm efficacy, elucidated the underlying mechanisms, and further investigated its synergistic antibacterial effects with fosfomycin sodium in both *in vitro* and *in vivo* models. The findings demonstrate that IFA effectively suppresses biofilm formation by specifically targeting the LuxS/AI-2 QS system, thereby reducing bacterial adhesion and motility, without exerting a direct bactericidal effect. Notably, IFA significantly potentiated the antibacterial activity of fosfomycin sodium against *E. coli*. In animal models, the combination therapy of IFA and fosfomycin exhibited superior therapeutic outcomes compared to fosfomycin monotherapy. This study not only identifies IFA as a novel anti-biofilm agent with a defined molecular target, offering new perspectives for combating antibiotic-resistant bacterial infections.

## 2 Materials and methods

### 2.1 Materials and bacterial strains

Isoferulic Acid, 4-Hydroxycoumarin, Daphnetin, Isoliquiritigenin, Carvacrol, α-Terpineol, Isofraxidin, Isoorientin, Isoquercitrin, Citronellol, Isophytol, Phlorizin, and Osthole used in this study was purchased from MedChemExpress. All compounds were dissolved in dimethyl sulfoxide (DMSO), with the final concentration of DMSO in the culture medium maintained at less than 1% (v/v). Fosfomycin sodium, cefquinome, gentamicin, polymyxin B, streptomycin, tetracycline, chloramphenicol, and azithromycin were purchased from MCE.

*E. coli* O157:H7 (ATCC 43895) was obtained from the American Type Culture Collection (ATCC). All *E. coli* strains were cultured in Luria-Bertani (LB) broth or on LB agar (Huankai Microbial, Guangdong, China), supplemented with antibiotics when necessary (ampicillin, Amp, 50 μg/mL). *Vibrio harveyi* (*V. harveyi*) BB170 and *V. harveyi* BB152 were generously provided by Dr. Han Xiang’an (Shanghai Veterinary Research Institute, Chinese Academy of Agricultural Sciences). These strains were maintained in autoinducer bioassay (AB) medium (The main components in each liter are 0.05 M MgSO_4_, 0.3 M NaCl, and 0.2% vitaminfree Casamino Acids) supplemented with 1 mM L-arginine, 1% phosphate buffer (pH 7.2), and 1% glycerol at 30 °C with shaking at 200 rpm.

The Caco-2 cell line was sourced from ATCC and cultured under standard conditions: Minimum Essential Medium (MEM) supplemented with 20% fetal bovine serum (FBS), 1 mM sodium pyruvate, 1 mM L-glutamine, 10 mM HEPES, and 1% non-essential amino acids.

Six-week-old female C57BL/6 J mice were purchased from the Lanzhou Veterinary Research Institute, Chinese Academy of Agricultural Sciences. All experimental procedures were strictly performed by the Chinese National Standard 《Requirements for Environment and Housing Facilities of Laboratory Animals》 (GB 14925-2001). The experimental protocol was approved by the Animal Welfare and Ethics Committee of the Lanzhou Institute of Husbandry and Pharmaceutical Sciences, Chinese Academy of Agricultural Sciences (Approval No.: 2025-16).

### 2.2 Construction and validation of the *Escherichia coli* LuxS-eGFP plasmid vector

The luxS gene fragment was PCR-amplified from *E. coli* genomic DNA using the specific primers luxS-F and luxS-R (Supplementary Table S1), with the pUC-19 plasmid serving as the backbone vector. Subsequently, the luxS fragment was fused in-frame with the enhanced green fluorescent protein (eGFP) gene via overlap extension PCR. The resulting luxS-eGFP fusion fragment was then cloned into the pUC-19 vector using restriction enzyme digestion and ligation with T4 DNA ligase, yielding the recombinant reporter plasmid pUC-19-LuxS-eGFP. Following construction, the plasmid was initially transformed into DH5α competent cells for amplification. After plasmid extraction, the construct was introduced into *E. coli* O157 competent cells via electroporation to generate the final LuxS-eGFP *E. coli* reporter strain.

For functional validation of the LuxS-eGFP reporter system, the recombinant *E. coli* strain was cultivated to mid-logarithmic phase (OD600 ≈ 0.6) and subsequently induced with 1 mM isopropyl β-D-1-thiogalactopyranoside (IPTG) for 4 h to drive the expression of the LuxS-eGFP fusion protein. The specificity and responsiveness of the biosensor were confirmed by challenging the induced culture with 60 μM Furanone C-30 (C30), a well-characterized quorum-sensing inhibitor targeting LuxS activity (Ren et al., 2001). In parallel, quantitative reverse transcription PCR (qRT-PCR) analysis was conducted to quantitatively assess the impact of C30 treatment on the transcriptional level of the endogenous luxS gene, thereby providing orthogonal validation at the mRNA level.

### 2.3 Screening of LuxS/AI-2 pathway inhibitors

The LuxS-eGFP *E. coli* reporter strain was cultured to mid-logarithmic phase (OD600 = 0.6) and subsequently induced with 1 mM isopropyl β-D-1-thiogalactopyranoside (IPTG) for 4 h to express the LuxS-eGFP fusion protein. Following induction, test compounds were administered at a final concentration of 400 μg/mL per compound. A known LuxS inhibitor, C30 (60 μM), was included as a positive control. The cultures were then subjected to an additional 4-h shaking incubation at 37 °C. Post-incubation, aliquots from each treatment group were transferred to a black 96-well microplate (Jingan, Shanghai, China). Fluorescence intensity was measured using a multifunctional microplate reader (Enspire, PerkinElmer, Waltham, MA, United States) with excitation wavelengths set at 488 nm and emission wavelengths set at 520 nm, respectively. Compounds demonstrating significant suppression of fluorescence were selected for further investigation.

### 2.4 AI-2 bioluminescence assay

The AI-2 bioluminescence assay was performed as previously described (Ali et al., 2018). Briefly, *E. coli* was cultured overnight in LB broth at 37 °C with shaking, while *Vibrio harveyi* BB170 was simultaneously grown in AB medium. The following day, bacterial cultures were subcultured (1:100) into fresh medium containing test compound and incubated for 10 h. Post-incubation, cell-free supernatants were obtained by centrifugation (12,000 × g, 5 min) followed by filtration through 0.22 μm sterile membranes. For the assay, *V. harveyi* BB170 cultures (pre-grown for 16 h) were diluted 1:5000 in fresh AB medium, and 90 μL of the diluted suspension was mixed with 10 μL of test supernatant in a 96-well plate. Following 4 h of incubation in the dark, bioluminescence was measured using a multifunctional microplate reader (Enspire, PerkinElmer, Waltham, MA, United States). All experiments were performed in triplicate.

### 2.5 The toxicity evaluation of IFA

#### 2.5.1 The effect of IFA on the growth and metabolic activity of *Escherichia coli*

The effect of IFA on *E. coli* growth was determined using the microdilution broth method. Serial dilutions of IFA (100, 200, 400 μg/mL) were co-incubated with standardized bacterial suspensions (OD_600_ = 0.01) in 96-well microplates at 37 °C for 24 h. Growth inhibition was quantified by measuring the optical density at 600 nm (OD₆₀₀) using a microplate reader (Thermo Fisher Scientific, United States).

The AB assay was employed to evaluate bacterial metabolic activity (Swetha et al., 2019). In brief, bacterial cultures were harvested by centrifugation, washed twice with phosphate-buffered saline (PBS, pH 7.4) to remove residual medium components, and subsequently resuspended in fresh, pre-warmed culture medium of equal volume to a standardized cell density. A 90 μL aliquot of bacterial suspension was dispensed into each well of a sterile 96-well microtiter plate. The reaction was initiated by adding 10 μL of the AB reagent solution to each well. The plate was then incubated for 1 h at 37 °C under light-protected conditions to prevent photobleaching of the fluorophore. Following incubation, the absorbance was measured at 570 nm (reduced form) and 600 nm (oxidized form) using a microplate reader. A blank control, consisting of PBS containing an equivalent concentration of AB reagent, was used for baseline correction. Metabolic activity was calculated according to Formula: Metabolic activity % = [Eoxi(_OD600_) × T_OD570_ − Eoxi(_OD570_) × T_OD600_] / [Ered(_OD570_) × B_OD600_ − Ered(_OD600_) × B_OD570_] × 100%, where Eoxi_(OD570)_ is the extinction coefficient in the oxidized form of AB at 570 nm = 80,586, Ered_(OD570)_ is the extinction coefficient in the reduced form of AB at 570 nm = 155,677, Eoxi_(OD600)_ is the extinction coefficient in the oxidized form of AB at 600 nm = 117,216, and Ered_(OD600)_ is the extinction coefficient in the reduced form of AB at 570 nm = 14,652. B represents blanks, and T represents samples.

#### 2.5.2 Cytotoxicity

The cytotoxic effects of IFA on Caco-2 cells were assessed using the Cell Counting Kit-8 (CCK-8) assay. Briefly, Caco-2 cells were seeded in 96-well plates at a density of 1 × 10^5^ cells/mL and cultured until the cell density reached approximately 80%. The cells were then treated with varying concentrations of IFA (25, 50, 100, 200, and 400 μg/mL) for 24 h. Following treatment, 10 μL of CCK-8 reagent (MedChemExpress, Shanghai, China) was added to each well, and the plates were incubated for an additional 1 h. Absorbance was measured at 450 nm using a microplate reader. Each experimental condition was assayed in triplicate to ensure reproducibility, and the entire procedure was independently repeated three times for robust statistical analysis.

### 2.6 Biofilm assay

#### 2.6.1 Biofilm inhibition assay

Biofilm formation was quantitatively assessed using the crystal violet (CV) staining method (Kvasničková et al., 2016). Briefly, an overnight culture of *E. coli* was prepared under static conditions at 37 °C, and the bacterial suspension was standardized to an optical density at 600 nm (OD600) of 0.01. Bacterial suspensions were mixed with varying concentrations of IFA (50, 100, 200, 400 μg/mL) in 96-well polystyrene plates (Corning Costar^®^ 3599, Corning, Kennebunk, ME, United States) and incubated statically for 24 h at 37 °C. Following incubation, planktonic cells were removed by washing three times with PBS. The adherent biofilms were then fixed with methanol and stained with 0.1% CV for 30 min. Unbound dye was removed by thorough rinsing with distilled water. Subsequently, the bound dye was eluted using 95% ethanol, and the absorbance of the resulting solution was quantified at 595 nm using a microplate spectrophotometer. All experiments were performed in triplicate.

#### 2.6.2 Biofilm eradication assay

*E. coli* was cultured under static conditions overnight at 37 °C. The bacterial suspension was subsequently standardized to an optical density at 600 nm (OD_600_) of 0.01. The bacterial suspension was then transferred to 96-well plates and incubated statically for 24 h at 37 °C to allow biofilm formation. Following incubation, non-adherent cells were removed by washing the biofilms three times with PBS. Subsequently, the biofilms were treated with various concentrations of IFA for 24 h. After treatment, the IFA solution was aspirated, and the biofilms were washed again with PBS. Excess stain was removed by thoroughly rinsing the wells with distilled water. The bound crystal violet was eluted using 95% ethanol, and the absorbance of the resulting solution was quantified spectrophotometrically at 595 nm. All experiments included three independent biological replicates.

#### 2.6.3 Scanning electron microscopy analysis

Biofilm architecture was examined using scanning electron microscopy (SEM) (Yang et al., 2024). Briefly, bacterial suspensions (OD_600_ = 0.01) supplemented with varying concentrations of IFA (100, 200, 400 μg/mL) were inoculated into 96-well plates pre-loaded with cell climbing slides, followed by incubation at 37 °C for 24 h to facilitate biofilm formation. The samples were gently washed three times with sterile PBS to remove planktonic bacteria. Subsequently, The adherent biofilms were then fixed and subjected to a graded ethanol series for dehydration. After sputter-coating with a thin layer of gold, the specimens were examined using an FEI Versa 3D SEM instrument (Thermo Fisher Scientific, United States). All experiments were performed with three independent replicates.

### 2.7 EPS production

The production of EPS was quantified using the ruthenium red staining method (Adnan et al., 2020). Briefly, bacterial suspensions (OD_600_ = 0.01) supplemented with varying concentrations of IFA (100, 200, 400 μg/mL) were dispensed into 96-well plates and cultivated at 37 °C for 24 h to facilitate EPS biosynthesis. After washing three times with PBS, 200 μL of 0.01% (w/v) ruthenium red solution was added to each well (with the blank control containing only the staining solution). The plates were subsequently incubated at 37 °C for 60 min under light-protected conditions. The residual staining solution was then transferred to a new 96-well plate, and the absorbance at 450 nm was measured using a microplate reader. The inhibition rate was calculated according to Formula 1:


$$\mathrm{EPS}\;\text{inhibitions}\;(\%)=\frac{\left(\mathrm{AS}-\mathrm{AP}\right)}{\left(\mathrm{AB}-\mathrm{AP}\right)}\times 100\%$$ (1)

AB: Absorbance of the blank control solution,

AS: Absorbance of the sample solution,

AP: Absorbance of the positive control (untreated *E. coli*) solution.

### 2.8 Motility assay

The effect of IFA on *E. coli* motility was assessed using a previously described method (Vikram et al., 2013). Briefly, an overnight bacterial culture was standardized to OD600 of 1.0. Subsequently, 1 μL of the suspension was spotted onto 0.3% semi-solid LB agar plates containing different concentrations of IFA (100, 200, 400 μg/mL). After incubation at 37 °C for 24 h, motility was evaluated by measuring the migration halo diameter (compared to the control group). All experiments included three independent biological replicates.

### 2.9 qRT-PCR

The qRT-PCR was performed to investigate the effect of IFA on the transcription of biofilm-regulated genes in *E. coli*. *E. coli* was cultured with or without IFA for 24 h at 37 °C. Total RNA was extracted using a Bacterial RNA Kit (Omega Bio-tek, Norcross, GA, United States). RNA concentration was measured using a NanoDrop OneC spectrophotometer (Thermo Scientific, United States), and RNA integrity was verified by agarose gel electrophoresis. Subsequently, cDNA was synthesized from RNA using the PrimeScript™ RT Reagent Kit with gDNA Eraser (Takara Bio, Kusatsu, Japan). qRT-PCR amplification was performed with TB Green^®^ Premix Ex Taq^™^ II (Tli RNaseH Plus) (Takara Bio) on a QuantStudio 6 Real-Time PCR System (Thermo Fisher Scientific, United States). The relative expression levels of target genes were normalized to the endogenous 16S rRNA gene and calculated using the comparative 2^−ΔΔCt^ method. The primer sequences used in this study are listed in Supplementary Table S2.

### 2.10 Antibacterial activity

The antibacterial activity of IFA was evaluated according to a previously described method (Liu et al., 2022). Briefly, overnight bacterial suspensions were diluted to an OD600 of 0.01 and then co-treated with different concentrations of antibiotics (1/2 MIC, 1/4 MIC, and 1/8 MIC) in the presence or absence of IFA (400 μg/mL). After incubation at 37 °C for 16–18 h, bacterial metabolic activity was determined using the AB assay.

### 2.11 Animal trial

Six-week-old female C57BL/6 J mice were acclimatized for 1 week prior to experiments. The mice were intraperitoneally inoculated with a suspension of *E. coli* O157:H7 (10^8^ CFU per mouse). The animals were then randomly allocated into five groups (*n* = 12 per group) and administered daily intraperitoneal injections of the following treatments: (i) Control (Received daily intraperitoneal injections of PBS), (ii) Modle (Inoculated with *E. coli* only, serving as the infection model), IFA (Inoculated with *E. coli* and treated daily with IFA (50 mg/kg) via intraperitoneal injection), (iii) fosfomycin sodium (Inoculated with *E. coli* and treated daily with fosfomycin sodium (200 mg/kg) via intraperitoneal injection), (iv) combination therapy of IFA and fosfomycin sodium (Inoculated with *E. coli* and treated daily with a combination of IFA (50 mg/kg) and fosfomycin sodium (200 mg/kg) via intraperitoneal injection). Survival rates were monitored and recorded daily throughout the 7-day treatment period. After 7 days of treatment, spleen, liver and ileum samples were collected for bacterial load quantification (via plate counting method). Additionally, some tissue samples were fixed in 4% paraformaldehyde solution for subsequent hematoxylin and eosin (H&E) staining and immunofluorescence staining of intestinal tight junction proteins (ZO-1 and Occludin).

### 2.12 Statistical analysis

Except for animal experiments, all other experiments were set up with three biological replicates, and all experiments were repeated three times. Student’s *t*-test was employed for comparisons between two groups, and one-way ANOVA followed by Tukey’s HSD test was used for comparisons involving three or more groups, with analyses conducted using GraphPad Prism 9.0 (GraphPad Software, San Diego, CA, United States). Data are shown as means ± SD. *p* < 0.05 was considered statistically significant.

## 3 Results

### 3.1 Validation of LuxS-eGFP *Escherichia coli* reporter strain

**Figure 1:**
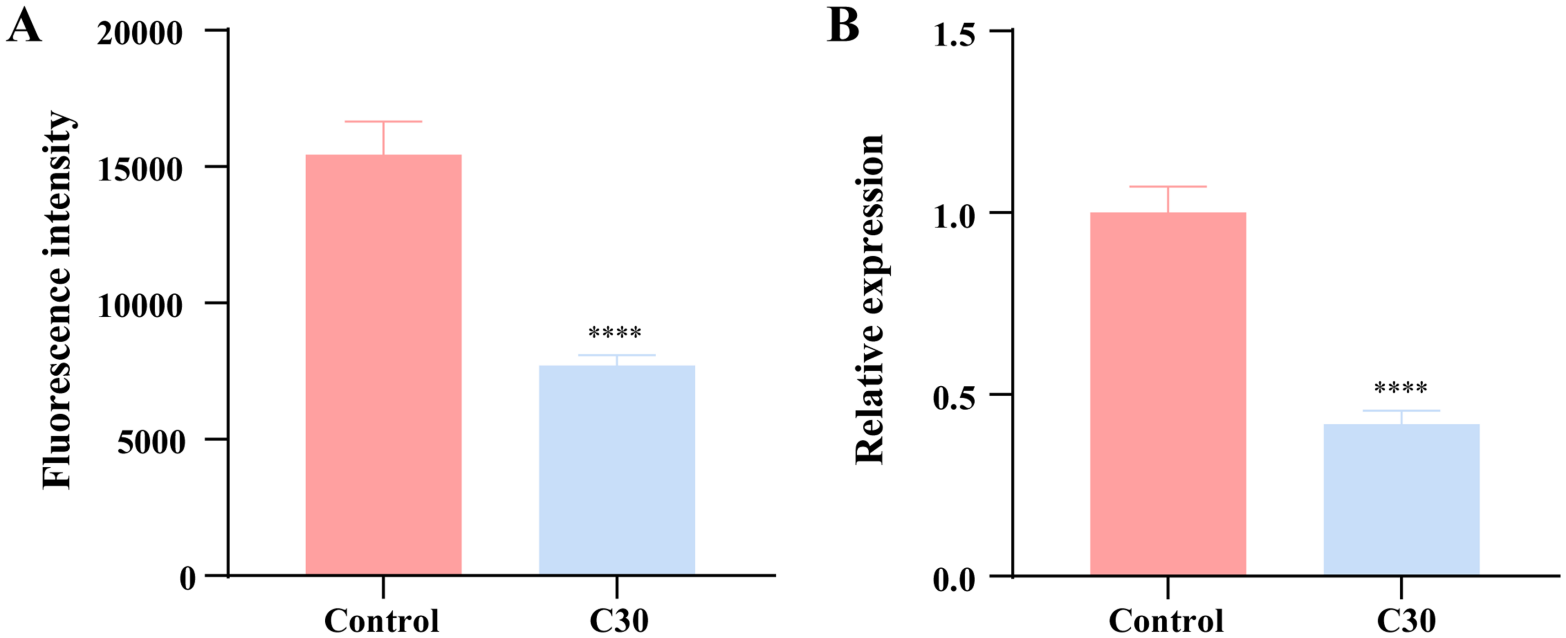
Validation of LuxS-eGFP *E. coli* reporter strain. **(A)** Effect of C30 on fluorescence intensity in LuxS-eGFP *E. coli*. **(B)** Effect of C30 on *luxS* gene expression in LuxS-eGFP *E. coli*. ****p < 0.0001.

The robustness and validity of the LuxS-eGFP reporter *E. coli* screening system was verified by employing C30, a well-characterized LuxS inhibitor. Results demonstrated that the LuxS-eGFP reporter strain exhibited strong fluorescent intensity. At the same time, C30 significantly inhibited both the fluorescence intensity (*Z*’ = 0.955) (Figure 1A) and *luxS* gene expression in the reporter strain (Figure 1B). These findings confirm the robustness and reliability of this model for subsequent screening applications.

### 3.2 Screening and evaluation of inhibitors for the LuxS/AI-2 quorum sensing

**Figure 2:**
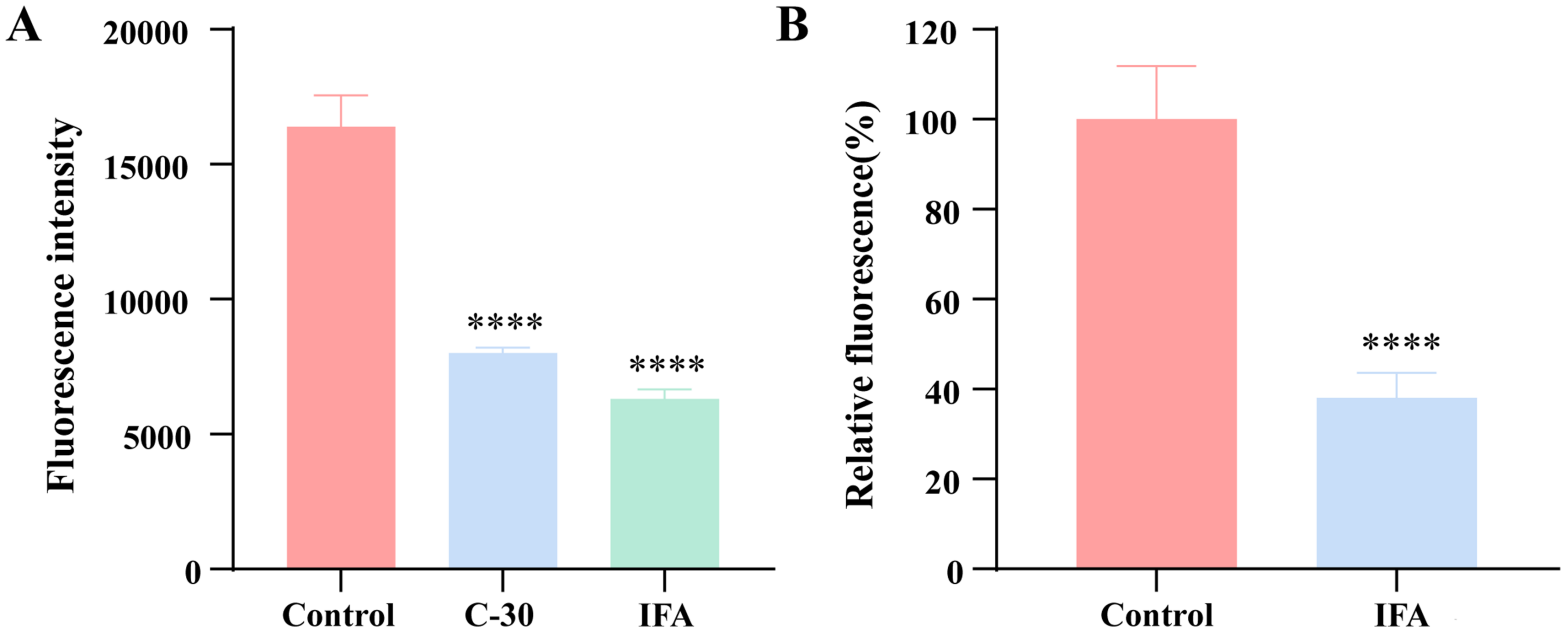
The effect of IFA on the *E. coli* LuxS/AI-2 quorum sensing system. **(A)** Impact of IFA on fluorescence intensity in LuxS-eGFP *E. coli*. **(B)** Effect of IFA treatment on AI-2 activity. ****p < 0.0001.

The LuxS-eGFP *E. coli* reporter strain was utilized to identify potent inhibitors of the LuxS/AI-2 QS system. As summarized in Supplementary Table S3, treatment with 400 μg/mL of isoferulic acid, 4-hydroxycoumarin, daphnetin, isoliquiritigenin, carvacrol, and α-terpineol led to a significant reduction in bacterial fluorescence intensity, with inhibition rates exceeding 50%. Notably, IFA demonstrated the most potent effect, achieving an inhibition rate of 63.43% (Figure 2A). Compounds with an inhibition rate greater than 50% were selected to determine their impact on the production of AI-2 in *E. coli*. The AI-2 bioluminescence assay revealed that isoferulic acid, isoliquiritigenin, carvacrol, and 4-hydroxycoumarin significantly attenuated LuxS-regulated AI-2 signal molecule production, as evidenced by inhibition rates surpassing 50% (Supplementary Table S2). Consistent with the fluorescence data, IFA again showed the strongest inhibitory activity, with an inhibition rate of 61.99% (Figure 2B). Based on its superior performance in both assays, IFA was selected for further mechanistic investigation.

### 3.3 The toxicity evaluation of IFA

**Figure 3:**
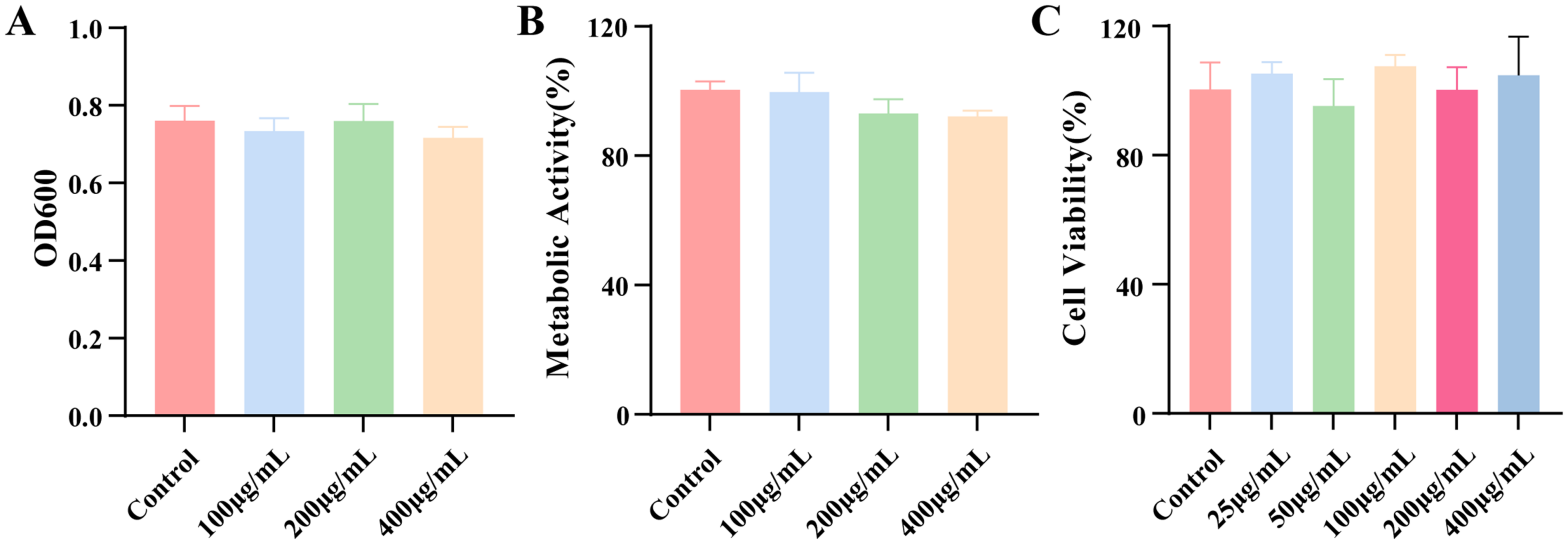
The toxicity evaluation results of IFA. **(A)** Growth activity of *E. coli* treated with different concentrations of IFA (100, 200, and 400 μg/mL). **(B)** Metabolic activity of *E. coli* treated with different concentrations of IFA (100, 200, and 400 μg/mL). **(C)** Cytotoxic effects of IFA on Caco-2 cells following 24-h treatment with different concentrations (25, 50, 100, 200, and 400 μg/mL).

Growth activity and metabolic activity of *E. coli* treated with different concentrations of IFA (100, 200, and 400 μg/mL) was evaluated using both microdilution broth method and AB assay. Compared with the control group, IFA at concentrations of 100, 200, and 400 μg/mL showed no inhibitory effects on either bacterial growth or metabolic activity (Figures 3A,B). Furthermore, the cytotoxicity profile of IFA was evaluated on Caco-2 cells. The results demonstrated that IFA exhibited no cytotoxicity at concentrations ≤400 μg/mL (Figure 3C). Furthermore, the cytotoxicity profile of IFA was evaluated on Caco-2 cells. Collectively, these results suggest that IFA lacks toxicity against both *E. coli* and host cells within the tested concentration range.

### 3.4 The effect of IFA on *Escherichia coli* biofilm

**Figure 4:**
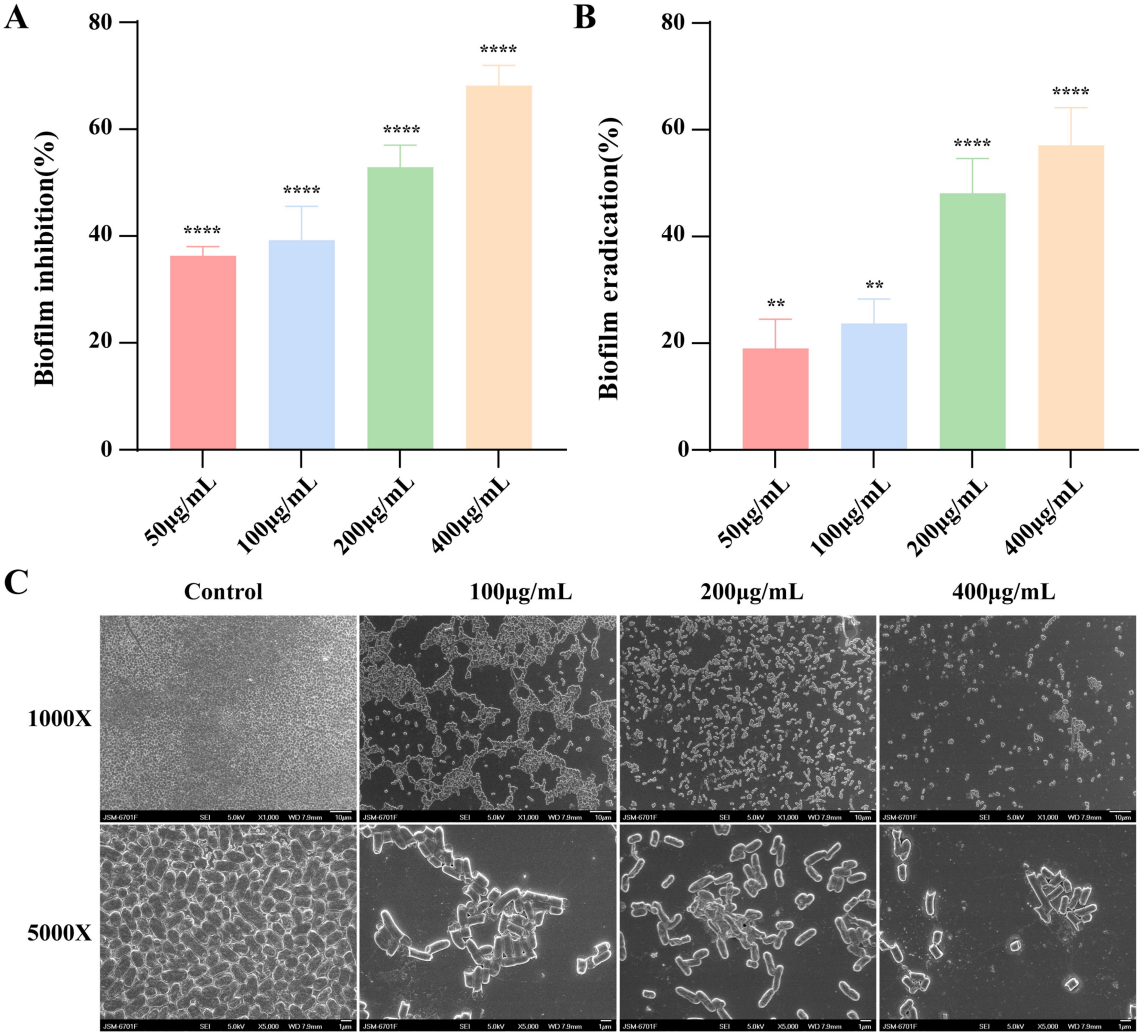
The effect of IFA on *E. coli* biofilms. **(A)** The inhibitory effect on biofilm formation after treatment with different concentrations of IFA (50, 100, 200, and 400 μg/mL) for 24 h. **(B)** The eradication capability of different concentrations of IFA (50, 100, 200, and 400 μg/mL) on mature biofilms. **(C)** SEM images (5,000×) of *E. coli* biofilms after treatment with different concentrations of IFA (100, 200, and 400 μg/mL) for 24 h. **p < 0.01, ****p < 0.0001.

The anti-biofilm activity of IFA was assessed using the CV staining method. Results from the biofilm inhibition assay demonstrated that IFA treatment significantly inhibited *E. coli* biofilm formation in a dose-dependent manner (Figure 4A). Notably, IFA effectively inhibited biofilm formation even at a concentration as low as 50 μg/mL. Furthermore, the biofilm eradication assay revealed that IFA could also significantly eliminate the preformed biofilm (Figure 4B). Scanning electron microscopy (SEM) imaging corroborated these findings, revealing a marked attenuation of biofilm architecture and density upon IFA exposure, thus providing visual validation of its potent anti-biofilm properties (Figure 4C).

### 3.5 Effects of IFA on EPS production of *Escherichia coli*

**Figure 5:**
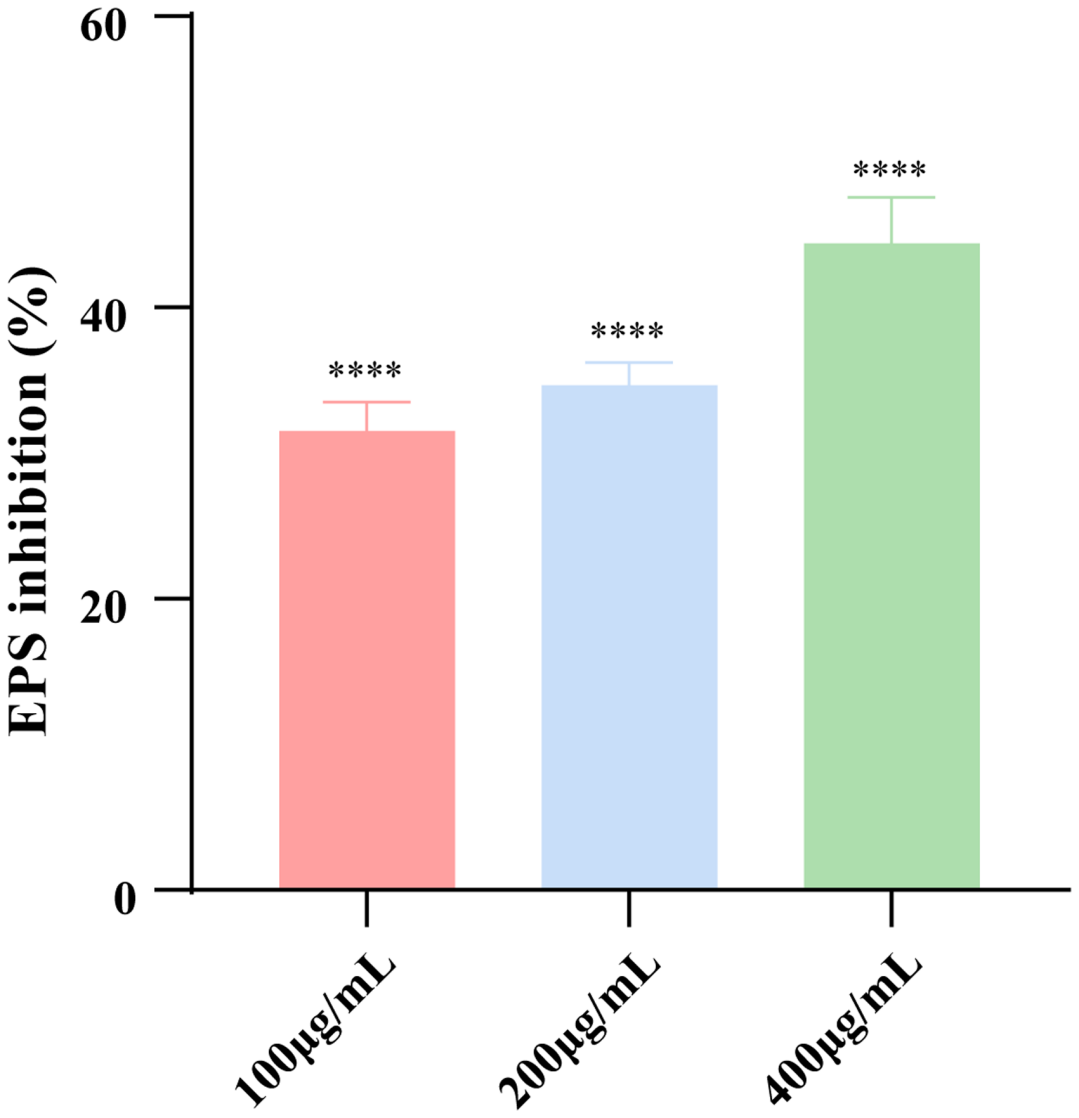
Effect of IFA on EPS inhibition. The inhibitory effects IFA on EPS production were evaluated at concentrations of 100, 200, and 400 μg/mL over a 24-h incubation period. ****p < 0.0001.

EPS form the fundamental architectural matrix of biofilms, playing a critical role in maintaining structural integrity and internal homeostasis. The production of EPS in biofilms was detected using the ruthenium red staining method, and the results showed that IFA significantly inhibited the production of EPS in *E. coli* in a dose-dependent manner (Figure 5).

### 3.6 Effects of IFA on the motility of *Escherichia coli*

**Figure 6:**
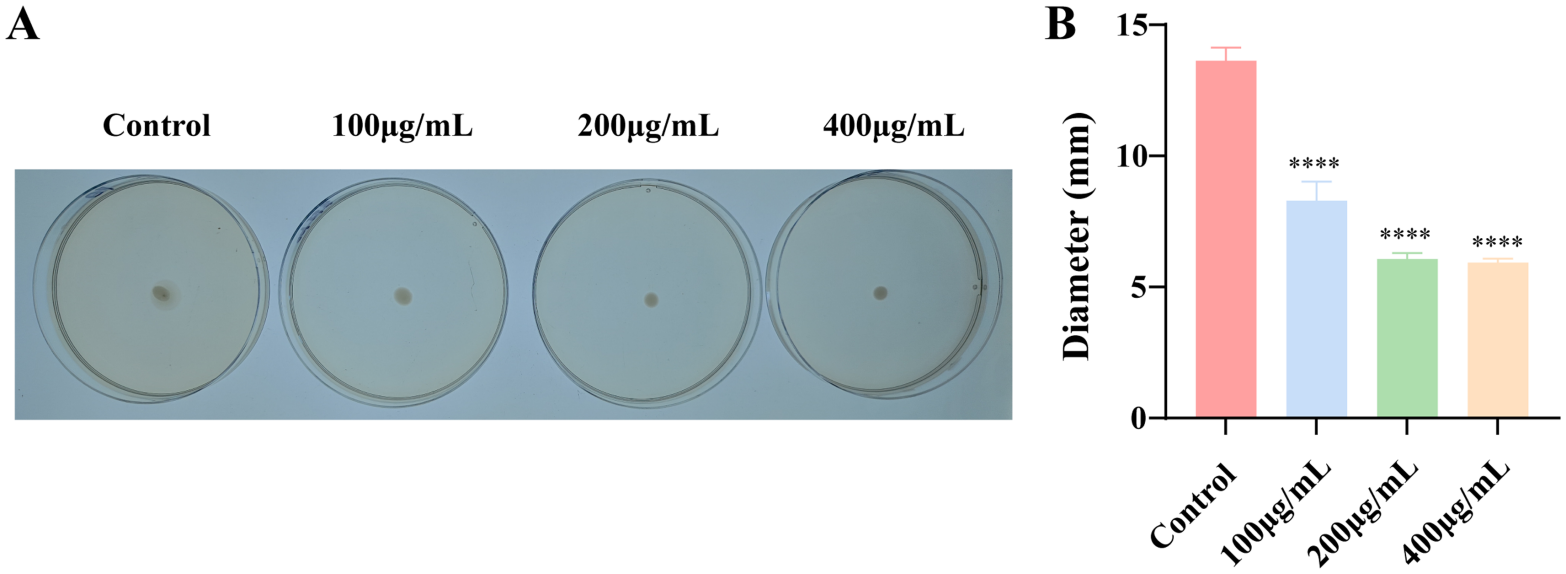
The effect of IFA on the motility of *E. coli*. **(A)** Images of the motility state of *E. coli* after co-culture with different concentrations of IFA (100, 200, and 400 μg/mL). **(B)** Quantitative analysis of motility based on the diameter of the diffusion halo. ****p < 0.0001.

A significant association was observed between bacterial motility and biofilm formation capacity. The experimental data demonstrated that IFA exerted a potent, dose-dependent inhibitory effect on the motility of *E. coli*, as evidenced by the marked reduction in bacterial migration compared to the vehicle control (Figure 6A). Quantitative assessment of the motility zone diameters further corroborated this finding, revealing statistically significant diminutions across all IFA-treated cohorts relative to the control group (Figure 6B).

### 3.7 Effect of IFA on the transcription of biofilm-regulated genes of *Escherichia coli*

**Figure 7:**
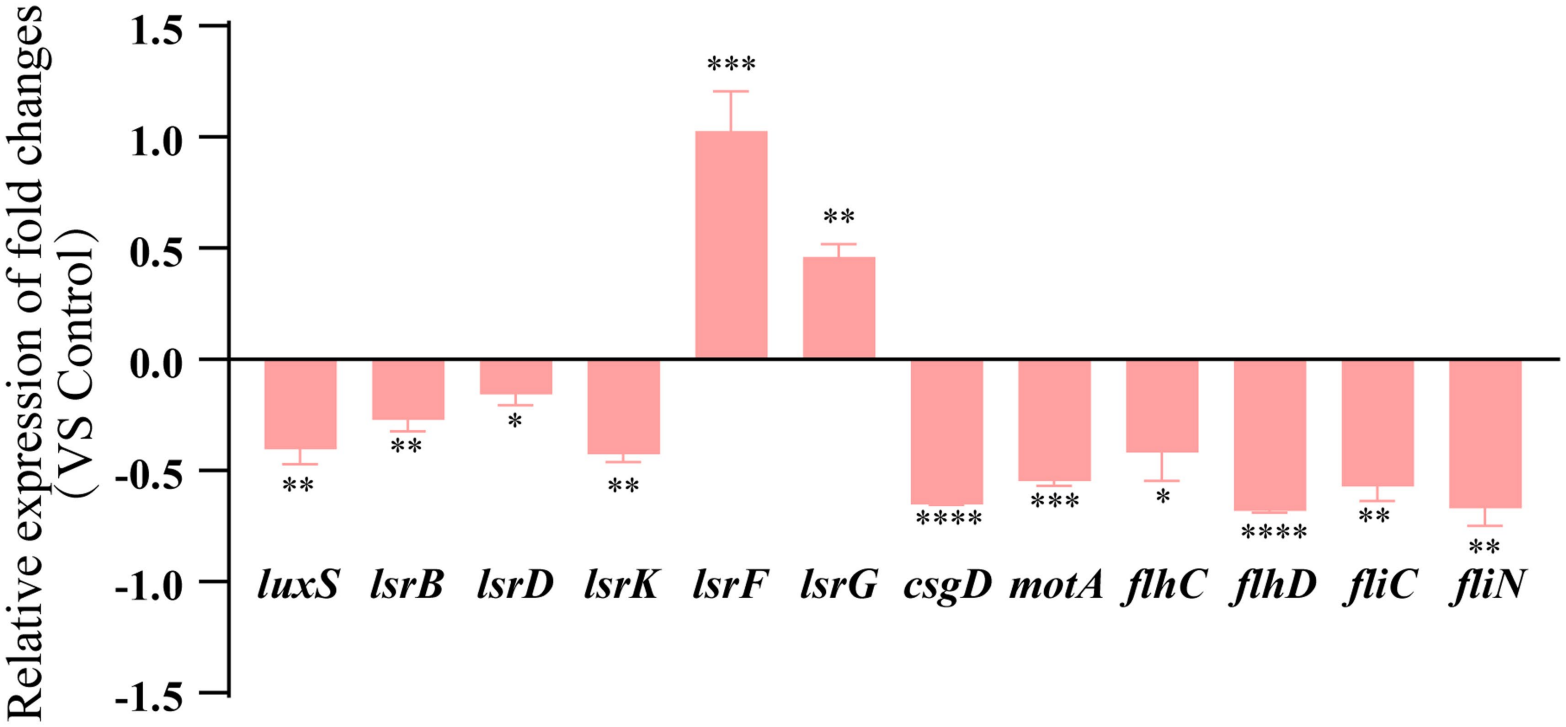
Effect of IFA on transcription of biofilm-related genes. qRT-PCR analysis revealed significant transcriptional alterations in 12 key genes (*luxS*, *lsrB*, *lsrD*, *lsrK*, *lsrF*, *lsrG*, *csgD*, *motA*, *flhC*, *flhD*, *fliC*, and *fliN*) compared to the control group. *p < 0.05, **p < 0.01, ***p < 0.001, ****p < 0.0001.

In this study, the effect of IFA on biofilm-related genes in *E. coli* was analyzed by qRT-PCR. The results showed that IFA could significantly differentially affect the expression of QS-related genes (*luxS*, *lsrB*, *lsrD*, *lsrK*, *lsrF*, *lsrG*), the curli fimbriae gene *csgD,* and motility-related genes (*motA*, *flhC*, *flhD*, *fliC*, and *fliN*) (Figure 7).

### 3.8 The synergistic effect of IFA combined with antibiotics against *Escherichia coli*

**Figure 8:**
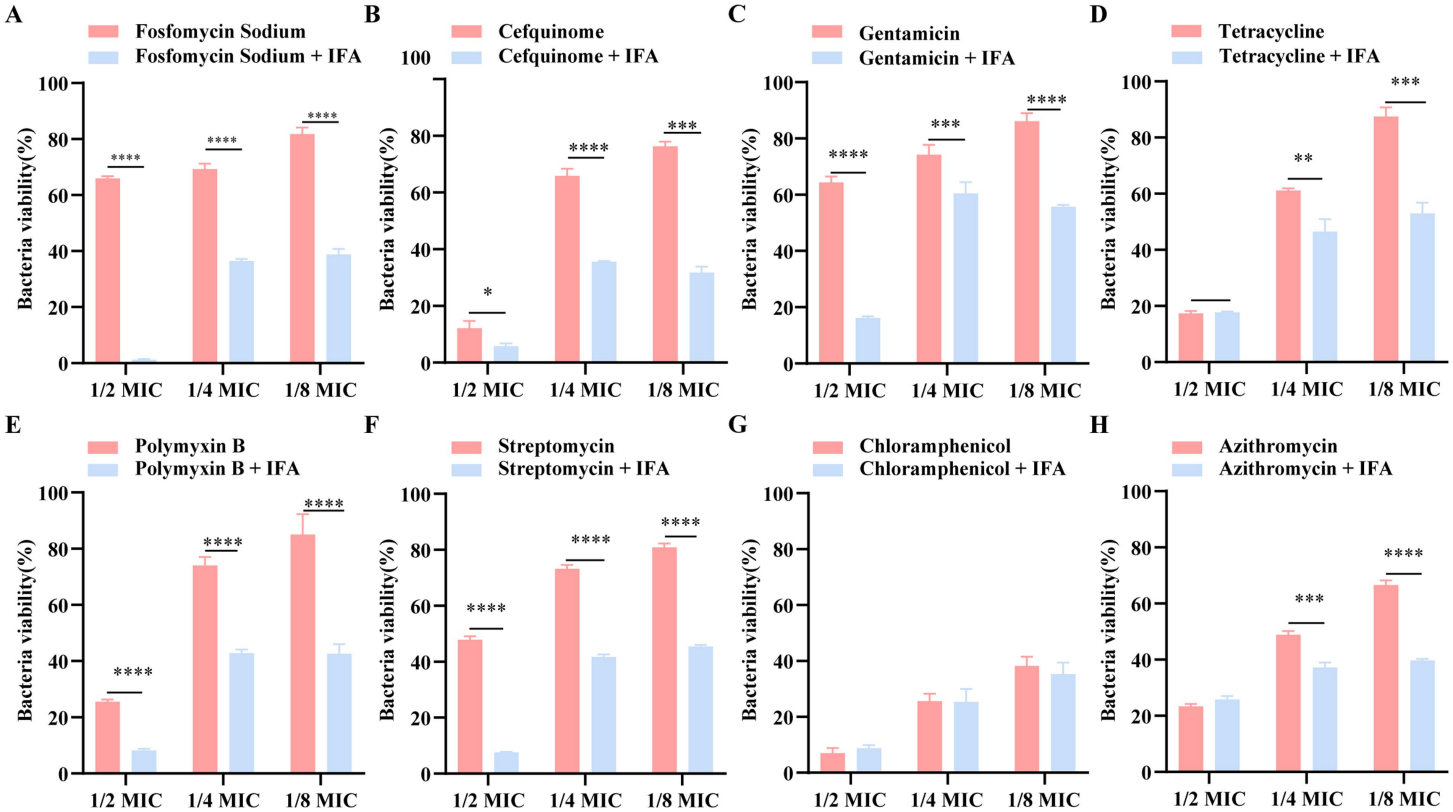
Effects of IFA (400 μg/mL) combined with antibiotics on bacterial viability of *E. coli* O157:H7. **(A)** Fosfomycin sodium. **(B)** Ceftriaxone. **(C)** Gentamicin. **(D)** Polymyxin B. **(E)** Streptomycin. **(F)** Chloramphenicol. **(G)** Tetracycline. **(H)** Azithromycin. *p < 0.05, **p < 0.01, ***p < 0.001, ****p < 0.0001.

The synergistic effect of IFA with antibiotics at concentrations of 1/2 MIC, 1/4 MIC, and 1/8 MIC was systematically evaluated through the AB assay. As shown in Figure 8, IFA significantly enhances the antibacterial effects of fosfomycin sodium (MIC = 128 μg/mL), cefquinome (MIC = 0.25 μg/mL), gentamicin (MIC = 0.5 μg/mL), polymyxin B (MIC = 0.5 μg/mL), streptomycin (MIC = 2 μg/mL), tetracycline (MIC = 2 μg/mL), chloramphenicol (MIC = 2 μg/mL), and azithromycin (MIC = 4 μg/mL) against *E. coli*. Notably, the most significant synergistic antibacterial activity was observed when IFA was combined with fosfomycin sodium. Specifically, the combination of IFA and 1/2 MIC fosfomycin sodium resulted in a substantial 64.69% reduction in bacterial metabolic activity compared to treatment with 1/2 MIC fosfomycin sodium alone. These findings suggest that the combination therapy of IFA with traditional antibiotics may represent a promising therapeutic strategy for treating biofilm-associated infections caused by pathogenic microorganisms such as *E. coli*.

### 3.9 *In vivo* evaluation of the IFA/fosfomycin sodium combination strategy

**Figure 9:**
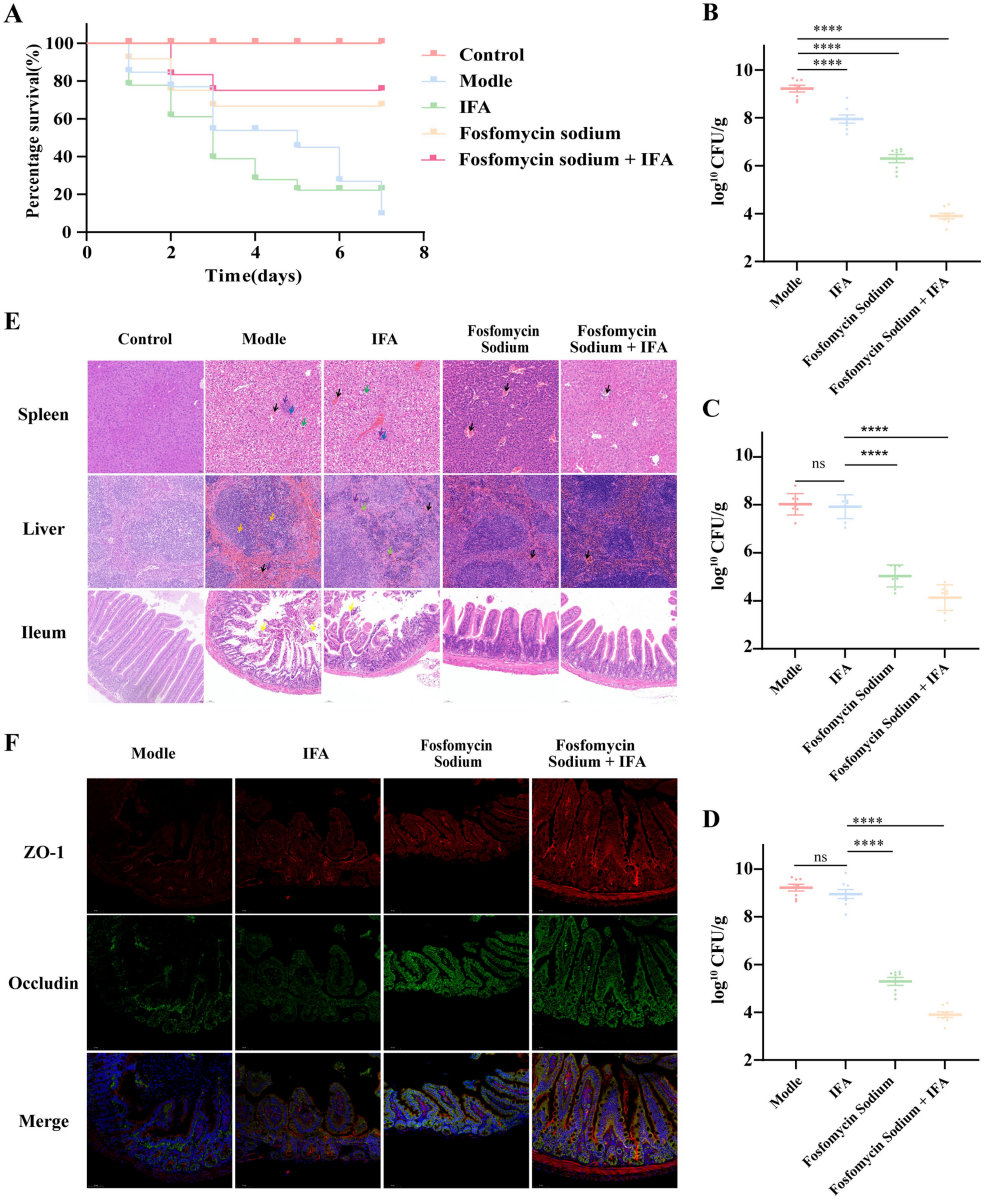
*In vivo* evaluation of the IFA/fosfomycin sodium combination strategy. **(A)** Mouse survival rate during the treatment period. **(B)** Bacterial load in ileum after different treatments. **(C)** Bacterial load in liver after different treatments. **(D)** Bacterial load in spleen after different treatments. **(E)** H&E staining of liver, spleen, and ileum tissues. Yellow arrows: shedding of intestinal villous epithelial cells, green arrows: cellular swelling, blue arrows: lymphocytes, purple arrows: granulocytes, black arrows: congestion, orange arrows: nuclear fragmentation, light green arrows: pigment deposition. **(F)** Fluorescence staining of tight junction proteins (ZO-1 and Occludin) in ileum tissues. ****p < 0.0001.

Based on preliminary experimental data, this study further evaluated the therapeutic effects of IFA combined with fosfomycin sodium on a mouse model infected with *E. coli* O157:H7. The combination regimen (50 mg/kg IFA + 200 mg/kg fosfomycin sodium) markedly improved the survival rate compared to either monotherapy (50 mg/kg IFA or 200 mg/kg fosfomycin sodium) (Figure 9A). Regarding bacterial clearance, he combination of IFA and fosfomycin sodium significantly reduced the bacterial load in the liver, spleen, and ileum (Figures 9B–D). Histological examination demonstrated severe pathological damage in the liver, spleen, and ileum of mice in the control and IFA-alone groups, whereas the combination therapy and fosfomycin sodium monotherapy groups exhibited only mild tissue injury (Figure 9E). Furthermore, immunofluorescence staining revealed that the expression levels of intestinal tight junction proteins (ZO-1 and Occludin) were significantly elevated in the combination therapy and fosfomycin sodium groups compared to the control and IFA groups, with the highest fluorescence intensity observed in the combination group (Figure 9F). Taken together, these data substantiate that the combination of IFA and fosfomycin sodium not only confers a survival benefit but also potentiates the antibacterial activity of fosfomycin sodium, thereby demonstrating a promising synergistic therapeutic effect.

## 4 Discussion

The formation of biofilms represents a critical obstacle in the management of clinical infections, particularly in cases involving recurrent chronic inflammation and medical device–associated infections (Bouhrour et al., 2024). Biofilms exhibit unique physical barrier properties and physiological traits that impede the penetration and efficacy of conventional antimicrobial agents, thereby prolonging therapeutic durations (Aleksandrowicz et al., 2023; Fuente-Nunez et al., 2023). Consequently, the development of effective biofilm inhibitors capable of mitigating or eradicating biofilms is of paramount clinical importance. The findings of the present study demonstrate that IFA effectively suppresses biofilm formation by specifically targeting the LuxS/AI-2 quorum sensing system and attenuating bacterial adhesion and motility, without exerting a significant impact on bacterial growth. Notably, IFA substantially enhances the antibacterial activity of fosfomycin sodium against *E coli in vitro* and *in vivo*. These results collectively suggest that IFA exhibits a dual therapeutic role against *E. coli*, functioning as both a potent anti-biofilm agent.

In natural environments and host organisms, the majority of bacteria exist in the form of biofilms (Elias and Banin, 2012), wherein EPS in the extracellular polymeric substances serve as a key structural and functional component governing biofilm formation and stability (Hayat et al., 2018; Wei and Ma, 2013; Ghafoor et al., 2011). Targeting the EPS matrix presents a dual therapeutic advantage: it not only impedes *de novo* biofilm formation but also compromises the structural integrity of established biofilms, thereby enhancing the penetration and efficacy of antimicrobial agents against embedded viable bacteria (Shen et al., 2016; Song et al., 2019; Han et al., 2022). Therefore, targeting EPS may serve as a potential strategy to disrupt intercellular communication and subsequently influence biofilm development. Supporting evidence includes the findings of Onbas et al., who reported that a cell-free extract of *Lactobacillus plantarum* F-10 attenuated bacterial drug resistance by reducing biofilm biomass, metabolic activity, and EPS content (Onbas et al., 2019). Similarly, Wang et al. demonstrated that metabolites from *Lactobacillus plantarum* inhibited *Bacillus licheniformis* biofilm formation on abiotic materials (stainless steel and glass) by suppressing EPS deposition (Wang et al., 2019). Furthermore, Fang Liu et al. elucidated that phenyllactic acid hindered *Enterococcus faecalis* biofilm formation by interfering with both cell migration and EPS production (Liu et al., 2020). Notably, as a natural phenolic acid compound, IFA has a structure similar to that of various known anti-biofilm phenolic acid derivatives, and existing studies have shown that such derivatives can exert anti-biofilm effects by interfering with the synthesis or structural stability of EPS (Xu et al., 2022). In line with these observations, our findings reveal that IFA exerts a dose-dependent, significant inhibition on EPS synthesis in *E. coli*, indicating that its anti-biofilm activity is mediated, at least in part, through the suppression of EPS biosynthesis.

The establishment of bacterial biofilms is critically dependent on quorum sensing (QS), a sophisticated cell-to-cell communication system that orchestrates collective behaviors to enhance bacterial fitness under adverse environmental conditions (Mukherjee and Bassler, 2019). Central to this process is the synthesis of the autoinducer-2 (AI-2) signal molecule, which is catalyzed by the key enzyme LuxS. In this study, we demonstrated that inhibition of the LuxS protein effectively suppresses AI-2 production. To exploit this mechanism, we engineered a LuxS-eGFP reporter strain and, by tracking the attenuation of fluorescence intensity, successfully identified IFA as a potent inhibitor targeting the LuxS/AI-2 pathway. Subsequent validation via qRT-PCR and AI-2 bioluminescence assays confirmed that IFA markedly downregulates *luxS* gene expression, leading to a substantial reduction in AI-2 generation. In *E. coli*, the Lsr system is responsible for the detection, uptake, and signal transduction of AI-2. When the Lsr system is affected, bacteria cannot effectively utilize AI-2. The qRT-PCR results of this experiment showed that IFA significantly downregulated the expression of *lsrB*, *lsrD*, and *lsrK*, and significantly upregulated the expression of *lsrF* and *lsrG*, which produce AI-2 degrading proteins. This renders the bacteria unable to effectively detect and utilize AI-2, thereby affecting biofilm formation. These results indicate that IFA can inhibit the biofilm formation ability of *E. coli* by affecting the LuxS/AI-2 pathway. This is consistent with the quorum sensing inhibitory mechanisms exhibited by isoferulic acid derivatives in other bacterial strains, further supporting the potential of phenolic acid analogs as quorum sensing inhibitors (Li et al., 2026). Additionally, we found that IFA significantly downregulated the expression of the curli fimbriae gene *csgD* and flagella-related genes (*motA*, *flhC*, *flhD*, *fliC*, and *fliN*), thereby inhibiting the adhesion and motility of *E. coli* and further suppressing biofilm formation. Taken together, our findings suggest that IFA exerts its anti-biofilm activity through a dual-pronged mechanism: (1) by disrupting the LuxS/AI-2 QS pathway, thereby diminishing both the production and functional utilization of the AI-2 signal, and (2) by attenuating bacterial surface adhesion and motility. The synergistic interplay of these mechanisms culminates in a potent inhibition of *E. coli* biofilm formation.

Biofilms constitute a formidable physical barrier that markedly diminishes the effective antibiotic concentration at the site of infection, elevating bacterial resistance by 100- to 1,000-fold and serving as a primary driver of chronic infections and therapeutic failure (Jakubovics et al., 2000; Venkatesan et al., 2015). In recent years, as biofilm infections caused by multidrug-resistant bacteria have become increasingly common, strategies utilizing natural active ingredients to inhibit or disrupt biofilms and enhance the efficacy of antibiotics have demonstrated unique advantages (Marchese et al., 2018; Huang et al., 2024; Samreen and Siddiqui, 2024). As natural phenolic acids, isoferulic acid and its derivatives have attracted extensive attention in this context. Previous studies have shown that such compounds not only possess inherent anti-biofilm properties, but also exert synergistic effects with various antibiotics, thereby reducing antibiotic dosage and mitigating the risk of drug resistance (Zhang et al., 2025). *In vitro* assessments conducted in this study revealed that IFA exhibits synergistic antibacterial activity with a panel of antibiotics, including sodium fosfomycin, cefquinome, gentamicin, polymyxin B, and streptomycin. Among these combinations, IFA paired with sodium fosfomycin demonstrated the most potent synergistic effect. Subsequent validation in a mouse infection model corroborated these findings. This synergy significantly amplified the *in vivo* antibacterial potency of fosfomycin sodium, confirming the translational potential of the IFA-antibiotic combination strategy. Collectively, these data substantiate the *in vivo* efficacy and safety of this approach, underscoring its viability for clinical application.

### 4.1 Limitations

There are several limitations in the present study. First, the *in vitro* findings may not fully translate to human clinical settings, and the *in vitro* results need to be further verified in more clinically relevant *in vivo* models. Second, the specific mechanism underlying the interaction between IFA and LuxS protein, including the exact binding site and kinetic characteristics, was not fully elucidated in this study. Third, the long-term safety profile of IFA in humans remains unknown and requires further systematic evaluation. In addition, only one *E. coli* O157:H7 strain was used in this study; thus, the broader applicability and generalizability of the conclusions need to be validated with more strains of different serotypes and origins. Finally, the mouse model employed intraperitoneal injection rather than the oral infection route, which is more relevant for this foodborne pathogen, and more appropriate infection models should be considered in future research.

## 5 Conclusion

This study elucidates that IFA exerts its anti-biofilm activity via a dual mechanism: (1) by specifically targeting the LuxS/AI-2 quorum sensing system, thereby markedly attenuating the production and functional efficiency of AI-2 signaling molecules; and (2) by potently suppressing bacterial surface adhesion and motility. The synergistic interplay between these two pathways culminates in a pronounced inhibition of *E. coli* biofilm formation. Notably, IFA demonstrates a robust synergistic effect with fosfomycin sodium, significantly potentiating the antibacterial efficacy of the latter in both *in vitro* and *in vivo* models. Collectively, these findings underscore the potential of IFA to serve as a anti-biofilm compound. Its co-administration with conventional antibiotics may offer a promising therapeutic strategy for the clinical management of biofilm-associated, drug-resistant *E. coli* infections.

## Data Availability

The original contributions presented in the study are included in the article/Supplementary material, further inquiries can be directed to the corresponding authors.
