# Supplementary material for: Isoferulic acid suppresses Escherichia coli biofilm formation via LuxS/AI-2 quorum sensing inhibition and synergizes with fosfomycin
Source: Front Microbiol. 2026 Jun 1;17:1837128. doi: 10.3389/fmicb.2026.1837128 (PMC13265544; doi:10.3389/fmicb.2026.1837128)
Supplement: Supplementary file 1 [file Table_1.DOCX]

**Supplementary Material**

**Table S1. The primer sequences of LuxS fragment amplification**

| **Gene** | **Sequence** |
| --- | --- |
| *luxS* -F | ggatcctctagaatgccgttgttagatagcttcacag |
| *luxS-* R | tgctcaccctccgatgtgcagttcctgcaacttctc |

| Primer Names | **Sequences**（5' to 3'） |
| --- | --- |
| *luxS*-F | GAAAACAATGAACACCCCGCATGG |
| *luxS*-R | TCCCTCTTTCTGGCATCACTTCTTTG |
| *lsrB*-F | AGTGCTGACCTGGGACTCTGATAC |
| *lsrB*-R | GCCATATCCACCAACATACCTCCTAAC |
| *lsrD*-F | GTTTCAAAGTGGTGTGCCGATGC |
| *lsrD*-R | AGATACAGCGTGCCAAGCGTAATC |
| *lsrK*-F | GATGAACCTACCGCCTCGCTTAC |
| *lsrK*-R | AACAATACCCACGCCAGTAGCAAG |
| *lsrF*-F | TCGGCAGCGAATATGAACATCAGTC |
| *lsrF*-R | CCATATCTTTGCCCACGCCAGTC |
| *lsrG*-F | AAGCCTATAAAGATGAAGACGCAGTGG |
| *lsrG*-R | CACGGCATCAAACCATTGAACAGAC |
| *csgD*-F | AATCGCTGGCAATTACAGG |
| *csgD*-R | CCGCTTCCATCATATCCAG |
| *motA*-F | GAAGCCTTGGAGCACTCTATCAACC |
| *motA*-R | CTTTGGTGTATTTGGAGCGACGAAAC |
| *flhC*-F | ATGCTGCCATTCTCAACCGACTG |
| *flhC*-R | CGCATCGACGCCATTACACAAAC |
| *flhD*-F | CGTTAGCGGCACTGACTCTTCC |
| *flhD*-R | TTGCGTCAACTGAGTAATCGTCTGG |
| *fliC*-F | TTACCAACCTGAACAACACCACTACC |
| *fliC*-R | ACATATTGGACACTTCGGTCGCATAG |
| *fliN*-F | CAATGGACGATCTGTGGGCTGAAG |
| *fliN*-R  16s RNA-F  16s RNA-R | CACCGCCAAATTGCTGGAACAC  GTGAAGTCATGCCAGGAGCT  CGAAGTATGCGTCCGGATCA |

**Table S2. Primer sequences for qRT-PCR analysis**

**Table S3 Screening results for inhibitors targeting the LuxS/AI-2 quorum sensing system.**

| **Compounds** | **inhibition rate(%)** |
| --- | --- |
| Isoferulic Acid | 63.43 |
| 4-Hydroxycoumarin | 62.27 |
| Daphnetin | 60.52 |
| [Isoliquiritigenin](https://www.medchemexpress.cn/Isoliquiritigenin.html) | 56.74 |
| Carvacrol | 55.83 |
| α-Terpineol | 55.19 |
| Isofraxidin | 44.21 |
| Isoorientin | 29.17 |
| Isoquercitrin | 21.95 |
| Citronellol | 7.55 |
| Isophytol | 7.38 |
| Phlorizin | 6.86 |
| Osthole | 3.68 |

**Table S4. Effect of inhibitors on AI-2 Production in *E. coli***

| Compounds | inhibition rate(%) |
| --- | --- |
| Isoferulic Acid | 61.99 |
| Isoliquiritigenin | 60.78 |
| Carvacrol | 57.3 |
| 4-Hydroxycoumarin | 51.22 |
| α-Terpineol | 48.15 |
| Daphnetin | 33.47 |


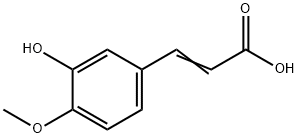


**Figure S1. The structural formula of isoferulic acid**
